# Stability analysis of the food delivery robot with suspension damping structure

**DOI:** 10.1016/j.heliyon.2022.e12127

**Published:** 2022-12-09

**Authors:** Shuhai Jiang, Wei Song, Zhongkai Zhou, Shangjie Sun

**Affiliations:** a School of Mechanical and Electronic Engineering, Nanjing Forestry University, Jiangsu, Nanjing, 210037, China; b Institute of Intelligent Control and Robotics (IICR), Nanjing Forestry University, Jiangsu, Nanjing, 210037, China

**Keywords:** Food delivery robot, Suspension structure, Dynamics, Stability, Zero moment point method

## Abstract

Aiming at the shock absorption and stability under complex terrain conditions, a six-wheeled food delivery robot with a suspension damping structure was designed. The food delivery robot is mainly composed of a take-out box, a chassis mobile structure and a suspension damping structure. The dynamic analysis of the suspension damping structure of the robot is carried out through the Lagrangian equation, and the offset of the wheels in different driving environments is obtained. Then, the zero-moment point method is used to analyze the stability of the food delivery robot in two non-horizontal movement environments of the left and right wheels and the front and rear wheels. Based on this, taking speed bumps and ramp terrain with different slopes as examples, the stability of the food delivery robot is analyzed by ADAMS simulation. The simulation and experiment results verify the rationality of the structure design of the food delivery robot with a suspension damping structure and the correctness of the theoretical analysis.

## 0 Introduction

The outbreak of COVID-19 has brought many inconveniences globally, and seriously affected all aspects of industrial production, transportation, and community life. With the emergence of different variants, limiting the spread of coronavirus has become much more difficult. In this general environment, in order to reduce the epidemic spread caused by the flow of people and dense gatherings, unmanned express delivery, unmanned food delivery, and unmanned logistics industries have gradually emerged, hence food delivery robots have been created. Food delivery robots are mobile robots, mainly used in high-density crowds such as universities, factories, and industrial parks, which deliver food with less safety hazards than that of manual food delivery. Wheeled mobile robots are the most common type of mobile robots. Common wheeled mobile systems include three-wheeled [1], [2], four-wheeled [3], [4], [5] and multi-wheeled [6], [7], [8], [9] mobile systems. When the robot has more than three wheels, suspension damping is required to ensure that all wheels are in contact with the ground. Therefore, the suspension damping structure is a very important part of the robot's mobile system. Based on the analysis of the existing multi-wheeled mobile robots' motion capability, the four-wheeled mobile system is the most common, with good motion stability, but its ability to overcome obstacles is limited; The five-wheel mobile system has an asymmetrical structure, and poor load carrying capacity; The six-wheel mobile system and the eight-wheel mobile system have strong terrain adaptability, carrying capacity and obstacle crossing ability [10], [11]. In order to realize the food delivery operation in the outdoor environment and ensure the stability and obstacle crossing performance, the six-wheel mobile system is used in the study. These kind of food delivery robots are different from other unmanned delivery robots, and takeaway meals are easy to dump and overflow during delivery. In order to avoid such a situation, the food delivery robot needs to have good shock absorption performance, that can ensure that it will not overturn when driving on various slopes, with a spring shock absorption structure. That is, it has good motion stability.

The suspension shock absorption structure [12], [13], [14], [15] is the main method for wheeled mobile robots to carry out obstacle reduction and improve terrain adaptability. The suspension structure is mainly divided into two parts: active adaptive suspension and passive adaptive suspension. The active adaptive suspension adapts to complex terrains by adjusting its own attitude, which is suitable for large and medium-sized unmanned vehicles; In comparison, the passive suspension structure is more suitable for smaller food delivery robots. Typical passive adaptive suspension structures include rocker-bogie suspension [16], [17], [18], parallelogram suspension [19], independent suspension [20], [21], and articulated rocker suspension [22], [23], [24]. In order to adapt to complex outdoor working environment, the existing suspension mechanism is comprehensively analyzed. The suspension structure is simplified under the premise of ensuring the mobile performance and stability of the robot, of which the dimensional parameters and spring stiffness are optimized to improve adaptability and obstacle crossing for the complex terrain.

Robot stability means that a robot in a balanced state is able to return to this state through a transition process when it is affected by external influences. That is, the smoothness of motion. Current stability analysis methods of robots mainly include static stability [25], [26] and dynamic stability [27], [28]. In 2012, Tharakeshwar Appala et al. [29] studied a three-wheeled mobile robot with a ring-shaped rear wheel, and used the force angle stability measurement technology to analyze and detect its overturning instability. In 2014, Zhong Guoliang et al. [30] proposed a new force angle stability margin (FASM) method, and evaluated the stability of three-wheeled robots and hexapod robots. In the same year, Akihiro Suzumura et al. [31], [32] introduced Zero Moment Point (ZMP) as a stability index improve the mobility of wheel-leg mobile robots, and studied the generation of whole body motion and various control systems. In 2019, Yu Suyang [33] et al. proposed a method to solve the ground reaction force using the motion state of the OMR, and used the calculated reaction force and force angular stability measurement (FASM) to determine the slip and tipping stability of the OMR did an evaluation. In the same year, Jiang Hui et al. [34] analyzed a passive-active transformation mobile robot, based on the evaluation method of the stability pyramid theory, and obtained an analytical expression for the relationship between the active suspension input and the cross-slope stability evaluation index.

With the suspension and shock absorption structure design, the robot has an imbalance in its own gravity in a complex terrain environment, which increases the angle difference between the left and right wheels and between the front and rear wheels of the robot in non-horizontal structures, thus reducing its stability. Therefore, it is necessary to obtain the relationship between the stability of the food delivery robot and the angle difference between the left and right wheels and the front and rear wheels of the robot in a non-horizontal structure, and the maximum slope angle that can maintain stability in both cases. In view of this, this article mainly designed a six-wheeled food delivery robot with a suspension damping structure, carried out dynamic modeling of the suspension structure, and analyzed the motion stability of the food delivery robot under different terrains. The simulation analysis is carried out based on ADAMS, and the simulation results verify the correctness of the structural design and theoretical analysis.

The authors of this paper designed a new six-wheel food delivery robot and analyzed its stability, mainly completing the following innovative work: A new outdoor food delivery robot with suspension damping structure was designed; The differential equation of motion of the suspension damping structure is obtained by using the Lagrange equation method, and the relationship between different spring rates and spring deformation is obtained; The zero-torque point method is used to analyze the stability of the food delivery robot in the non-horizontal attitudes of the left and right wheels and the non-horizontal attitudes of the front and rear sides. The displacement caused by the suspension damping structure of the food delivery robot when the body is non-horizontal is substituted into the stability analysis of the robot, which increases the authenticity and accuracy of the stability analysis of the food delivery robot; The displacement and speed curve of the food delivery robot through the speed reduction belt under different spring stiffness were studied, and multiple sets of simulation experiments were carried out on the food delivery robot with different slopes and different spring shock absorber stiffness. The difference between the slope angle of different slopes and the length change of the front and rear wheel spring shock absorbers when the robot overturned was also obtained.

This paper is divided into 5 parts, Section 0 is the introduction, which gives the background and necessity of the thesis research; Section 1 gives the overall structure and suspension structure design of the robot, and gives the dynamic analysis of the suspension structure; Section 2 discusses the stability analysis of the robot, mainly introduces the stability analysis method, the non-horizontal motion stability analysis of the left and right vehicles, and the non-horizontal stability analysis of the front and rear wheels; Section 3 gives the simulation and experiment analysis, mainly discusses the stability analysis of the robot when passing the speed bump, and the stability analysis of the robot when climbing; Section 4 presents the results and discussion of the paper.

## 1 The overall structure design of the robot

### 1.1 Overall structure design

**Figure 1:**
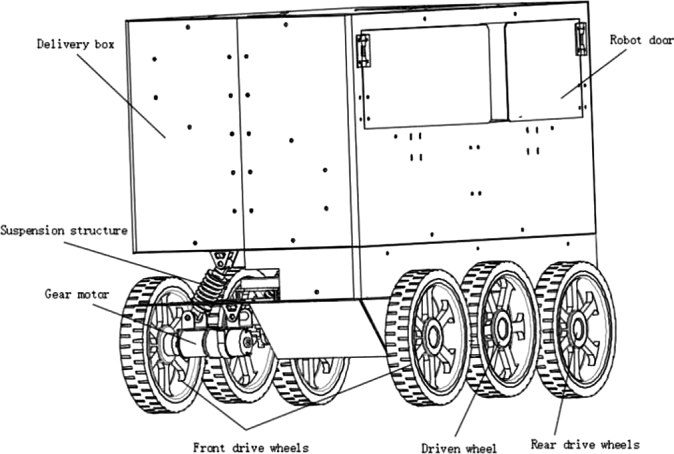
The overall structure of the food delivery robot.

As shown in Fig. 1, the overall structure of the food delivery robot is composed of a robot box, a chassis drive structure, and a suspension damping structure. The food delivery robot is a six-wheel mobile system. The six wheels are symmetrically distributed on both sides of the food delivery robot, which increases the stability and running performance of the food delivery robot. The six wheels are equipped with drive motors and reducers. The food delivery robot adopts a differential driving turning method, and the left and right sides drive the wheels in forward and reverse directions or the differential speed to control the robot to turn. A suspension damping structure is installed between the geared motor of the food delivery robot and the robot support chassis. The spring system of the suspension damping structure is used to achieve the effect of damping. The suspension damping structure is symmetrically fixed on both sides of the mobile robot chassis structure. There are three pairs of six suspension damping structures.

### 1.2 Suspension structure design

**Figure 2:**
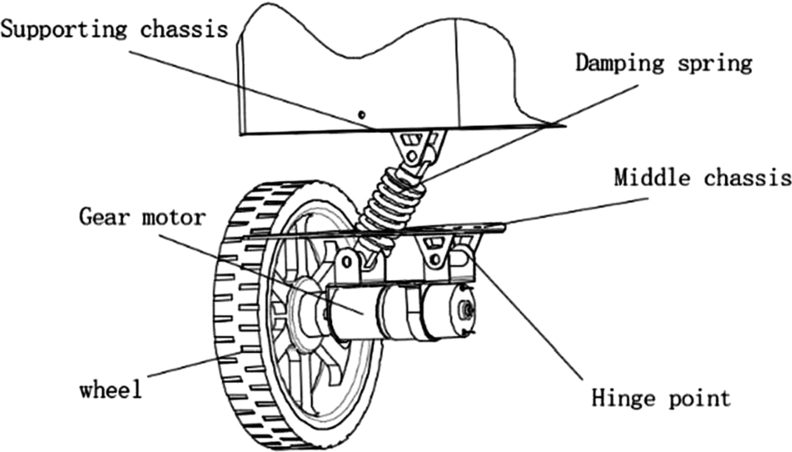
The structure diagram of the independent suspension of the food delivery robot.

The suspension damping structure is the key structure of the wheeled mobile robot, which determines the obstacle-crossing and damping performance of the mobile robot on uneven roads. Fig. 2 is a three-dimensional structure diagram of the independent suspension structure of the food delivery robot. It includes four main structures: shock-absorbing spring, geared motor, middle chassis and supporting chassis. The shock-absorbing spring, the geared motor, the middle chassis, and the supporting chassis are hinged together by long bolts to form a four-bar-like structure. When the wheel passes the bumpy ground or a speed bump, the motor and the frame as a whole rotate around the hinge point of the middle chassis, which drives the damping spring to compress upwards, hence relieving the damping effect and increasing the motion stability of the robot.

### 1.3 Dynamic analysis of suspension damping structures

In the process of movement, the food delivery robot with suspension shock absorption structure needs to consider the impact of the displacement of the suspension shock structure in the vertical direction on the stability of the robot. Hence it is necessary to establish a dynamic model of the robot suspension shock absorption structure to analyze the relationship between the external impact force of the wheel and the displacement of the wheel in the vertical direction of the food delivery robot in the case of non-horizontal movement, laying the foundation for the stability analysis of the food delivery robot. In the design process of the food delivery robot, it is necessary to select and design the spring stiffness of the suspension shock absorption structure, and to perform dynamic analysis of the suspension shock absorption structure to obtain the relationship between the spring rate and the spring change, which provides a theoretical basis for the selection of the spring stiffness. Since the food delivery robot adopts a symmetrical structure design and is an independent suspension structure, regarding studying the relationship between the external impact force on the wheel and the displacement of the wheel in the vertical direction, it is only necessary to study the dynamic characteristics of the shock absorption structure of one single suspension, thus simplifying the modeling process and improving the efficiency of the study.

**Figure 3:**
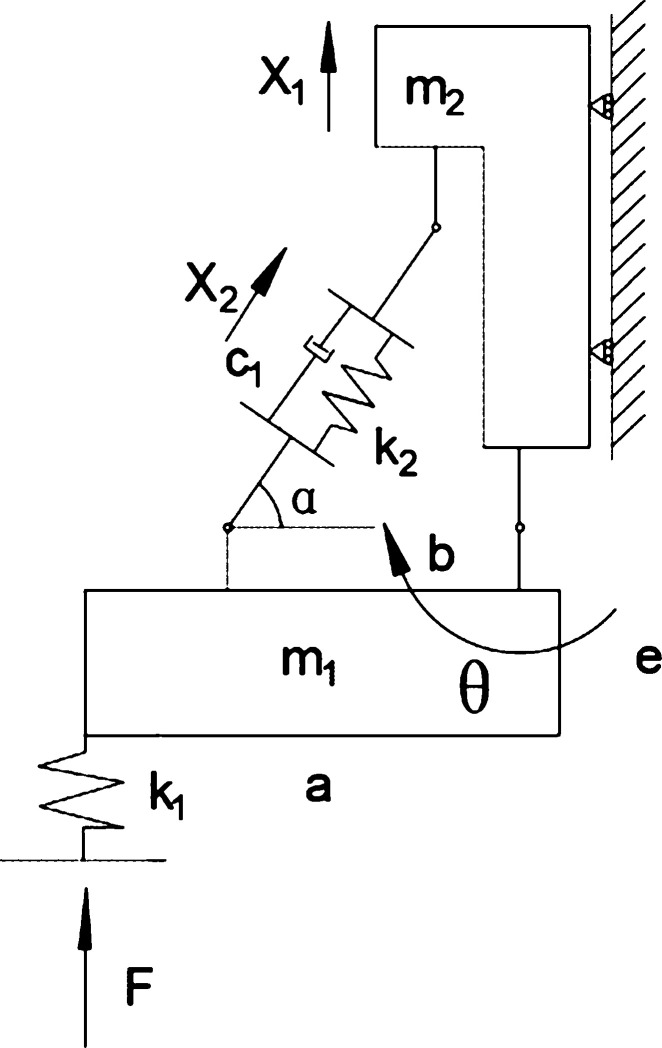
Stress analysis diagram of single independent suspension structure after conversion.

As shown in Fig. 3, a mechanical diagram of a single independent suspension structure for a food delivery robot. In the figure, the $c_{1}$ is the drag coefficient of the spring shock absorber, the $k_{2}$ is the spring stiffness of the spring shock absorber; the angle between the spring shock absorber and the horizontal direction when the *α* is the initial state; the $m_{1}$ is the weight of the food delivery robot driving the motor and the reducer, and the $m_{2}$ is one-sixth of the weight of the feeding robot; the $k_{1}$ is the elastic stiffness of the robot wheel tire; the $X_{1}$ is the displacement of the body in the vertical direction, the $X_{2}$ is the compression in the direction of the shock absorbing spring, and the *θ* is the angle of the driving motor and the reducer around the fixed hinge point *F* is the support force and impact force of the tire on the ground, and *e* is the distance from the center of mass of the driving motor to the center of the shaft. Because the wheel tires are rubber solid tires, the deformation generated by them is much smaller than the deformation of the entire suspension structure, so the relatively small influence of tire deformation on displacement is ignored.

The generalized coordinates of the establishment system are:

(1) $$q=\left[\begin{array}{c} X_{1}\\ X_{2} \end{array}\right]$$

According to formula (1) and Fig. 3, total kinetic energy of the system:

(2) $$T=\frac{1}{2b^{2}}I{\overset{˙}{X_{2}}}^{2}\sin^{2}\alpha +\frac{1}{2b^{2}}m_{1}(\frac{a^{2}}{4}+e^{2}){\overset{˙}{X_{2}}}^{2}\sin^{2}\alpha +\frac{1}{2}m_{1}{\overset{˙}{X_{1}}}^{2}+\frac{1}{2}m_{2}{\overset{˙}{X_{1}}}^{2}+\frac{1}{2}m_{2}{\overset{˙}{X_{2}}}^{2}\sin^{2}\alpha$$

In the formula, *I* is the moment of inertia of the drive motor and the reducer around the articulation point.

According to Fig. 3, the virtual work of the system is:

(3) $$l_{W}=[F-m_{1}g-\frac{2\mathrm{Fa}-m_{1}ga}{2b}-m_{2}g+(k_{2}X_{2}-c_{1}{\overset{˙}{X}}_{2})\sin\alpha ]l_{X_{1}}+[\frac{2\mathrm{Fa}-m_{1}ga}{2b\sin\alpha }-(k_{2}X_{2}-c_{1}{\overset{˙}{X}}_{2})]l_{X_{2}}$$

Then, according to formula (3), the generalized forces of the system are:

(4) $$Q_{1}=\frac{l_{W}}{l_{X_{1}}}{|}_{l_{X_{1}}\neq 0,\;l_{X_{2}}=0}=F-m_{1}g-m_{2}g+(k_{2}X_{2}-c_{1}{\overset{˙}{X}}_{2})\sin\alpha$$

(5) $$Q_{2}=\frac{l_{W}}{l_{X_{2}}}{|}_{l_{X_{1}}=0,\;l_{X_{2}}\neq 0}=\frac{2\mathrm{Fa}-m_{1}ga}{2b\sin\alpha }-(k_{2}X_{2}-c_{1}{\overset{˙}{X}}_{2})$$

Therefore, according to formula (4) and (5), the expression for generalized force *Q* is:

(6) $$Q=\left[\begin{array}{c} Q_{1}\\ Q_{2} \end{array}\right]=\left[\begin{array}{c} F-m_{1}g-\frac{2\mathrm{Fa}-m_{1}ga}{2b}-m_{2}g+(k_{2}X_{2}-c_{1}{\overset{˙}{X}}_{2})\sin\alpha \\ \frac{2\mathrm{Fa}-m_{1}ga}{2b\sin\alpha }-(k_{2}X_{2}-c_{1}{\overset{˙}{X}}_{2}) \end{array}\right]=\left[\begin{array}{cc} 0\; & k_{2}\sin\alpha \\ 0\; & -k_{2} \end{array}\right]\left[\begin{array}{c} X_{1}\\ X_{2} \end{array}\right]+\left[\begin{array}{cc} 0\; & -c_{1}\sin\alpha \\ 0\; & c_{1} \end{array}\right]\left[\begin{array}{c} {\overset{˙}{X}}_{1}\\ {\overset{˙}{X}}_{2} \end{array}\right]+\left[\begin{array}{c} F-m_{1}g-\frac{2\mathrm{Fa}-m_{1}ga}{2b}-m_{2}g\\ \frac{2\mathrm{Fa}-m_{1}ga}{2b\sin\alpha } \end{array}\right]$$

According to the Lagrange equation:

(7) $$\frac{d}{dt}(\frac{\partial T}{\partial {\overset{˙}{q}}_{k}})-\frac{\partial T}{\partial q_{k}}=Q_{k},\;k=1,2.$$

According to formula (2) and (7), where

(8) $$\frac{d}{dt}(\frac{\partial T}{\partial {\overset{˙}{q}}_{k}})=\left[\begin{array}{c} (m_{1}+m_{2}){\overset{¨}{X}}_{1}\\ \frac{I\sin^{2}\alpha }{2b^{2}}{\overset{¨}{X}}_{2}+\frac{1}{2b^{2}}m_{1}(\frac{a^{2}}{4}+e^{2})\sin^{2}\alpha {\overset{¨}{X}}_{2}+m_{2}{\overset{¨}{X}}_{2}\sin^{2}\alpha \end{array}\right]=\left[\begin{array}{cc} m_{1}+m_{2}\; & 0\\ 0\; & \frac{I\sin^{2}\alpha }{2b^{2}}+m_{2}\sin^{2}\alpha +\frac{1}{2b^{2}}m_{1}(\frac{a^{2}}{4}+e^{2})\sin^{2}\alpha \end{array}\right]\left[\begin{array}{c} {\overset{¨}{X}}_{1}\\ {\overset{¨}{X}}_{2} \end{array}\right]$$

(9) $$\frac{\partial T}{\partial q_{k}}=0$$

Substituting equations (6), (8), and (9) into the Lagrange equation (7) yields the dynamic equations for the suspension damping structure as follows:

(10) $$\left[\begin{array}{cc} m_{1}+m_{2}\; & 0\\ 0\; & \frac{I\sin^{2}\alpha }{2b^{2}}+m_{2}\sin^{2}\alpha +\frac{1}{2b^{2}}m_{1}(\frac{a^{2}}{4}+e^{2})\sin^{2}\alpha \end{array}\right]\left[\begin{array}{c} {\overset{¨}{X}}_{1}\\ {\overset{¨}{X}}_{2} \end{array}\right]\;=\left[\begin{array}{c} F-m_{1}g-\frac{2\mathrm{Fa}-m_{1}ga}{2b}-m_{2}g+(k_{2}X_{2}-c_{1}{\overset{˙}{X}}_{2})\sin\alpha \\ \frac{2\mathrm{Fa}-m_{1}ga}{2b\sin\alpha }+(k_{2}X_{2}-c_{1}{\overset{˙}{X}}_{2}) \end{array}\right]$$

$$M=\left[\begin{array}{cc} m_{1}+m_{2}\; & 0\\ 0\; & \frac{I\sin^{2}\alpha }{2b^{2}}+m_{2}\sin^{2}\alpha +\frac{1}{2b^{2}}m_{1}(\frac{a^{2}}{4}+e^{2})\sin^{2}\alpha \end{array}\right]$$

$$K=\left[\begin{array}{cc} 0\; & k_{2}\sin\alpha \\ 0\; & -k_{2} \end{array}\right]$$

$$C=\left[\begin{array}{cc} 0\; & -c_{1}\sin\alpha \\ 0\; & c_{1} \end{array}\right]$$

$$F_{c}=\left[\begin{array}{c} F-m_{1}g-\frac{2\mathrm{Fa}-m_{1}ga}{2b}-m_{2}g\\ \frac{2\mathrm{Fa}-m_{1}ga}{2b\sin\alpha } \end{array}\right]$$

namely:

(11) $$M\overset{¨}{q}=C\overset{˙}{q}+Kq+F_{c}$$

Then, the differential equation of motion for the suspension damping structure is:

(12) $$M\overset{¨}{q}-C\overset{˙}{q}-Kq-F_{c}=0$$

After obtaining the differential equation (10)–(12) of the suspension shock absorption structure, the length of the spring is obtained by USING MATLAB software to obtain the change of the length of the spring when subjected to different ground impact forces, because in the process of movement, the situation of being subjected to the sinusoidal force is more common, therefore, the simulation experiment is carried out under the influence of the sine force of the robot to obtain the length change of different spring stiffness.

The sinusoidal forces are:

(13) F=40+20sin⁡2t

**Figure 4:**
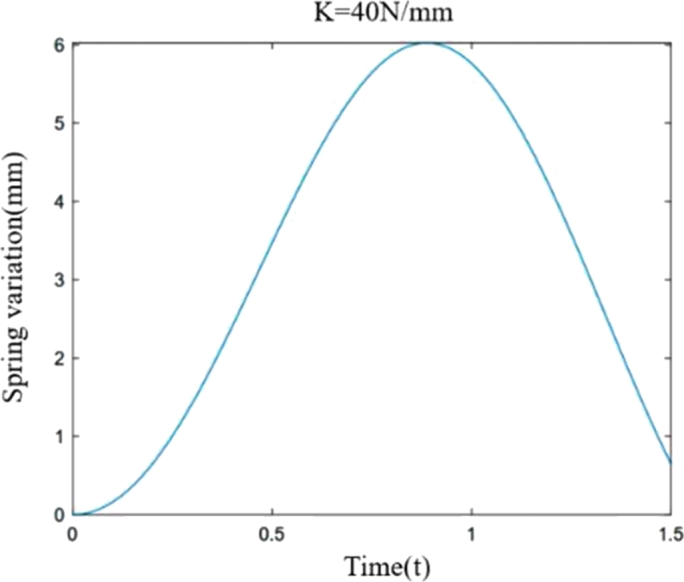
Variation of spring stiffness 40 N/mm and spring length.

**Figure 5:**
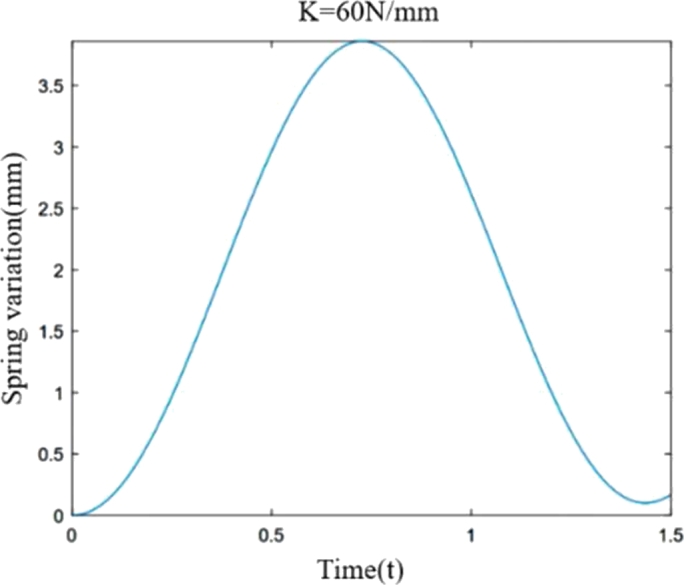
Variation of spring stiffness 60 N/mm and spring length.

**Figure 6:**
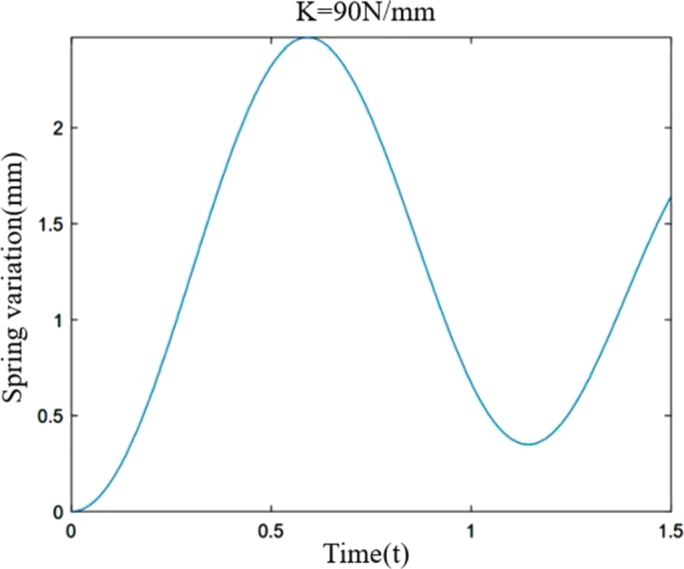
Variation of spring stiffness 90 N/mm and spring length.

According to formula (13), the parameters of the food delivery robot are substituted into the differential equation of motion, and the spring length variation curve is drawn according to the different spring stiffness as shown in Figure 4, Figure 5, Figure 6(From MATLAB).

Figure 4, Figure 5, Figure 6 show the spring variations of the suspension shock structure at stiffness of 40 N/mm, 60 N/mm and 90 N/mm respectively when the ground impact force of the wheel tire is sinusoidal force. Except the stiffness, the other data parameters are the same. Through comparison, it is found that when subjected to the same ground impact force, the greater the stiffness of the spring, the smaller the peak it reaches, and the shorter the time it requires to reach the peak. When the spring rate is 40 N/mm, 60 N/mm and 90 N/mm, the value of the spring change is 6.025 mm, 3.86 mm and 2.467 mm respectively. That is, under the same action, the greater the spring stiffness, the smaller the amount of deformation. Through the dynamic analysis of the shock absorption structure of a single suspension, the motion differential equation of the single suspension shock absorption structure is established, which provides theoretical support and reference for the stiffness selection design of the food delivery robot, and a theoretical basis for the stability analysis of the food delivery robot.

Specially, when the food delivery robot moves at a uniform speed on the flat ground without impact force, *F* is only the support force of the ground, that is, $F=m_{1}g+m_{2}g$, the acceleration and speed of the shock absorption structure of the food delivery robot are 0, that is:

(14) $$\left[\begin{array}{c} {\overset{¨}{X}}_{1}\\ {\overset{¨}{X}}_{2} \end{array}\right]=\left[\begin{array}{c} {\overset{˙}{X}}_{1}\\ {\overset{˙}{X}}_{2} \end{array}\right]=0$$

So, according to formula (14), equation (10) can be simplified to:

(15) $$\left[\begin{array}{cc} 0\; & k_{2}\sin\alpha \\ 0\; & -k_{2} \end{array}\right]\left[\begin{array}{c} X_{1}\\ X_{2} \end{array}\right]+\left[\begin{array}{c} F-m_{1}g-\frac{2\mathrm{Fa}-m_{1}ga}{2b}-m_{2}g\\ \frac{2\mathrm{Fa}-m_{1}ga}{2b\sin\alpha } \end{array}\right]=0$$

According to formula (15), after simplification, when the food delivery robot moves at a uniform speed, the amount of change in the spring is:

(16) $$\left[\begin{array}{c} X_{1}\\ X_{2} \end{array}\right]=\left[\begin{array}{c} 0\\ \frac{2\mathrm{Fa}-m_{1}ga}{2bk_{2}\sin\alpha } \end{array}\right]$$

According to the geometric relationship of the suspension shock absorption structure, the combined formula (16), the displacement formula (17) of the wheels of the suspension shock absorption structure in the vertical direction can be obtained:

(17) $$x=\frac{a}{b}X_{2}\sin\alpha =\frac{a^{2}}{2b^{2}k_{2}}(2F-m_{1}g)$$

Through the dynamic analysis of the suspension shock absorption structure, the motion differential equation of the suspension shock absorption structure is obtained. The spring deformation under different spring stiffness conditions is then calculated by MATLAB, which provides a certain theoretical reference for the selection and design of the spring stiffness of the food delivery robot. A model of the relationship between the external impact force of the wheel and the displacement of the wheel in the vertical direction under the non-horizontal movement of the food delivery robot is then established, which provides a theoretical basis for the stability analysis of the suspension shock absorption structure. The stability impact brought by the deformation of the suspension shock absorption structure is substituted into the stability analysis.

## 2 Stability analysis of food delivery robots

### 2.1 Stability analysis methods

At present, the evaluation index of robot stability is divided into static stability and dynamic stability discrimination index. Static stability refers to the ability of the system to change when a small disturbance occurs, and the ability of returning to the original state of stable operation when the disturbance disappears. Dynamic stability refers to the ability of the system to maintain the operational stability of a longer process under the action of automatic regulation and control devices after being subjected to small or large disturbances [35]. Common static stability methods include the center of gravity projection method (CGPM) [36], the static stable boundary method (SSM) [37] and the energy stabilization boundary method (ESM) method [38]. Common dynamic stability determination methods include the zero-force moment point method (ZMP) [39], [40], the pressure center method (COP), the force-angle method (FASM) [41], [42], [43], and the Poincaré mapping-Lyapunov criterion method [44]. Different determination methods are suitable for different operating environments, such as flat and sloped ground, flat and smooth ground and uneven ground, etc., which have an important impact on the selection of stability determination methods.

Takeaway food delivery robots will encounter a variety of complex sports environments during the delivery process, such as relatively large potholed terrain, hills and laterally along ramps. By summarizing the various outdoor driving environments, the posture of the food delivery robot mainly can be divided into two situations. One is that the left and right wheels are non-horizontal postures, such as turning on the sloped road surface, etc. while the other is that the front and rear wheels are non-horizontal, such as common climbing and speed bumps. Considering that, the force on the corresponding suspension shock absorption structure will be different when the wheels are not horizontal, combined with the dynamics of the suspension shock absorption structure in the previous section, the stability analysis of the two attitudes of the takeaway delivery robot is carried out.

### 2.2 Non-horizontal stability analysis of the left and right wheels

Through the comprehensive analysis of the existing stability determination method, the zero moment point method (referred to as ZMP) is used to analyze the stability of the wheeled mobile robot, and the ZMP point and stability margin value of the food delivery robot are obtained by combining the spring suspension structure of the food delivery robot and the angle of the front and rear wheels. The ZMP method was proposed by Vukobratovic in 1968, which holds that if there are gravitational forces, external forces and inertial forces on the ground, the point with a combined moment of 0 at the point is called a zero moment point. If the ZMP is in the support area, the robot is stable.

**Figure 7:**
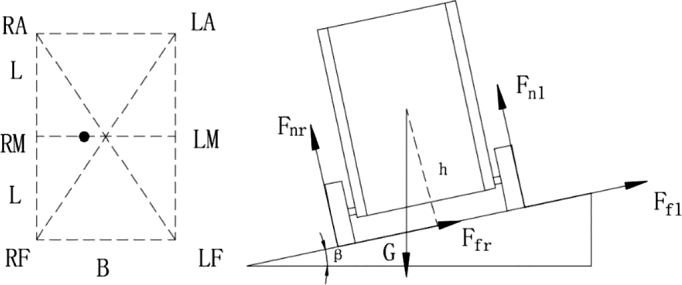
Stress analysis diagram of each wheel when the left and right wheels move non horizontally.

As shown in Fig. 7, for the force analysis of the lateral movement of the food delivery robot on the slope, 2*L* is the distance between the front and rear drive wheels of the takeaway delivery robot and the ground contact point; the $F_{nr}$ is the sum of the support forces of the right wheel facing the ground; the $F_{fr}$ is the sum of the friction forces of the ground facing the right wheel; the $F_{nl}$ is the sum of the support forces of the ground face of the left wheel; the $F_{fl}$ is the sum of the friction forces of the ground facing the left wheel; *h* is the vertical height of the robot's center of gravity to the ground, and *B* is the width distance of the robot's left and right wheels. Define the contact points of the six wheels to the ground as the left front wheel (*LF*), left center wheel (*LM*), left rear wheel (*LA*), right rear wheel (*RA*), right center wheel (*RM*) and right front wheel (*RF*).

From Fig. 7, the force analysis is obtained:

(18) $$\left\{ \begin{array}{c} F_{nl}\cdot B+h\cdot G\sin\beta =\frac{B}{2}\cdot G\cos\beta \\ F_{nr}\cdot B=h\cdot G\sin\beta +\frac{B}{2}\cdot G\cos\beta \end{array}\right.$$

According to formula (18), the supporting forces of the left and right wheels are:

(19) $$\left\{ \begin{array}{c} F_{nl}=\frac{1}{2}\cdot G\cos\beta -\frac{h\cdot G\sin\beta }{B}\\ F_{nr}=\frac{1}{2}\cdot G\cos\beta +\frac{h\cdot G\sin\beta }{B} \end{array}\right.$$

According to formula (19), since the robot is driving laterally, the supporting force of the same side of the wheel is the same, and the supporting force of each wheel is:

(20) $$\left\{ \begin{array}{c} F_{nlf}=F_{nlm}=F_{nla}=\frac{1}{6}\cdot G\cos\beta -\frac{h\cdot G\sin\beta }{3B}\\ F_{nrf}=F_{nrm}=F_{nra}=\frac{1}{6}\cdot G\cos\alpha +\frac{h\cdot G\sin\beta }{3B} \end{array}\right.$$

After the dynamic analysis of the suspension structure of the food delivery robot, the amount of change in the shock absorption structure of the robot suspension running smoothly on the flat road was *x*, and *F* in formula (17) was the combined force of the supporting force and the impact force of the robot on the ground. When the robot runs smoothly on a flat road, ignoring the impact force caused by the bumps on the ground, *F* at this time is the support force of the robot. Substitute type (20) (17) obtains the displacement bias of the suspension shock absorption structure of the robot moving laterally on the slope:

(21) $$\left\{ \begin{array}{c} x_{l}=\frac{a^{2}}{2b^{2}k_{2}}\left(\frac{1}{3}\cdot G\cos\beta -m_{1}g-\frac{2h\cdot G\sin\beta }{3B}\right)\\ x_{r}=\frac{a^{2}}{2b^{2}k_{2}}\left(\frac{1}{3}\cdot G\cos\beta -m_{1}g+\frac{2h\cdot G\sin\beta }{3B}\right) \end{array}\right.$$

Set $\beta^{\prime }$ is the angle between the left and right wheels and the horizontal plane of the food delivery robot under the influence of the spring shock absorption structure, when the food delivery robot travels laterally on the slope, the angle of the left and right wheels relative to the horizontal plane is the slope angle of the slope *β* and the tilt angle caused by the suspension structure Δ*β* sum, that is:

(22) $$\beta^{\prime }=\beta +\Delta \beta$$

According to the formula (21), that is, the displacement bias of the front and rear wheels of the spring damping mechanism $x_{l}$ and $x_{r}$, it is possible to obtain:

(23) $$\sin\Delta \beta =\frac{2ha^{2}G\sin\beta }{3b^{2}k_{2}B^{2}}$$

In the suspension shock structure, because the offset of the wheels moving up and down is much smaller than the width of the car, the value of the Δ*β* is smaller, so the approximate is equal to:

(24) Δβ=sin⁡Δβ

According to formula (22)–(24), so:

(25) $$\beta^{\prime }=\beta +\frac{2ha^{2}G\sin\beta }{3b^{2}k_{2}B^{2}}$$

**Figure 8:**
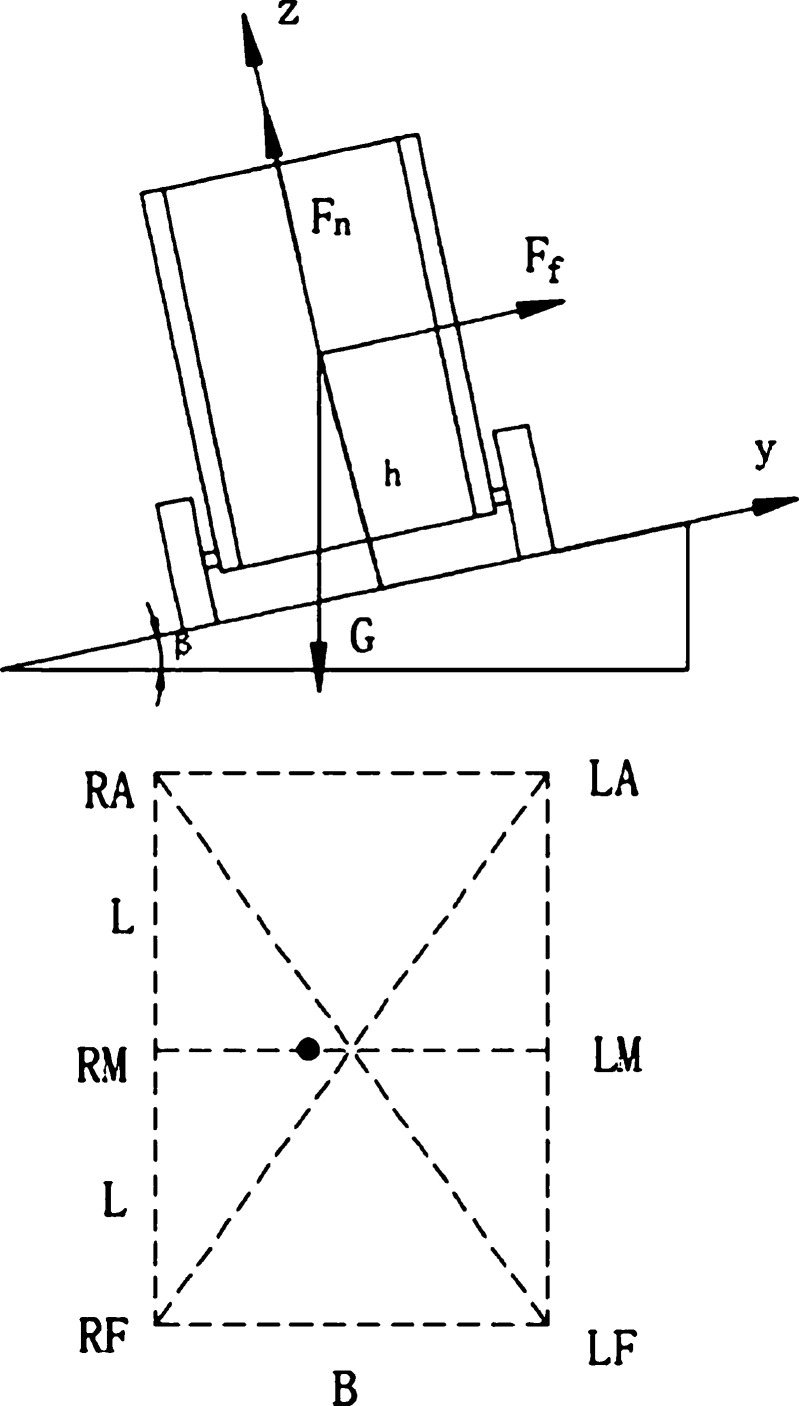
ZMP determination method when left and right wheels are not horizontal.

In order to determine the coordinate position of the support polygon and the ZMP point, the global coordinate system is established with the projection point of the center of mass perpendicular to the slope of the feeding robot as the origin of the coordinate system, and the x-axis is pointed to the forward direction of the robot, the z-axis is perpendicular to the oblique face, and the y-axis is determined by the right-hand rule. As shown in Fig. 8, the gravitational acceleration is set to $g=[0-g\sin\beta^{\prime }-g\cos\beta^{\prime }]$, $\beta^{\prime }$ is the angle between the left and right wheels and the horizontal plane of the feeding robot under the influence of the spring-loaded shock structure, and the center of gravity coordinate is $p_{c}=[x_{c}\;y_{c}\;z_{c}]$; *p* point is the position of the ZMP, the coordinate is $p=[x_{p}\;y_{p}\;z_{p}]$, and the support point is *f* by the ground reaction force; the moment *τ* of the ground reaction force around the origin is:

(26) $$\tau =p\cdot f+\tau_{p}$$

In the formula: $\tau_{p}$ is the torque at the zero crossing moment point.

Derived from the differential form of the momentum theorem and the angular momentum theorem:

(27) $$\frac{dP}{dt}=F=Mg+f$$

(28) $$\frac{dL}{dt}=M=p_{c}\times Mg+\tau$$

According to formula (27) and (28), namely:

(29) $$\overset{˙}{P}=Mg+f$$

(30) $$\overset{˙}{L}=p_{c}\times Mg+\tau$$

In the syndic (26), (29) and (30), eliminate *τ* and *f* to obtain:

(31) $$\tau_{p}=\overset{˙}{L}-p_{c}\times Mg+(\overset{˙}{P}-Mg)\times p$$

To rewrite formula (31) in the form of a component quantity:

(32) {τpx=L˙x+yc⋅Mgcos⁡β′−zc⋅Mgsin⁡β′τpx=+(P˙y+Mgsin⁡β′)zp−yp(P˙z+Mgcos⁡β′)τpy=L˙y−xc⋅Mgcos⁡β′+xp(P˙z+Mgcos⁡β′)−zpP˙xτpz=L˙z+xc⋅Mgsin⁡β′−xp(P˙y+Mgsin⁡β′)+P˙xyp

From ZMP theory, the horizontal components $\tau_{px}$ and $\tau_{py}$ in the above equation are 0, and the ZMP position coordinates are solved $x_{p}$ and $y_{p}$ are:

(33) $$\left\{ \begin{array}{c} x_{p}=\frac{x_{c}\cdot Mg\cos\beta^{\prime }+{\overset{˙}{P}}_{x}z_{p}-{\overset{˙}{L}}_{y}}{{\overset{˙}{P}}_{z}+\mathrm{Mg}\cos\beta^{\prime }}\\ y_{p}=\frac{{\overset{˙}{L}}_{x}+y_{c}\cdot Mg\cos\beta^{\prime }-z_{c}\cdot Mg\sin\beta^{\prime }+({\overset{˙}{P}}_{y}+Mg\sin\beta^{\prime })z_{p}}{Mg\cos\beta^{\prime }+{\overset{˙}{P}}_{z}} \end{array}\right.$$

The takeaway delivery robot is a quality point system, so its momentum and angular momentum are:

(34) $$\left\{ \begin{array}{c} P=\sum_{i}P_{i}=\sum_{i}m_{i}\overset{˙}{p_{i}}\\ L=\sum_{i}L_{i}=\sum_{i}p_{i}\times m_{i}\overset{˙}{p_{i}} \end{array}\right.$$

In the formula: $P_{i}$ and $L_{i}$ are the momentum and angular momentum of the *i* particle; $m_{i}$ is the mass of the *i* particle; $p_{i}$ is the coordinate position of the *i* particle.

Takeaway food delivery robot in the delivery of food for a uniform speed, when the robot is moving on the slope at a uniform speed, in order to ensure the safety of the takeaway food delivery robot, there is only straight movement, that is, no turning movement, ignoring the influence of the surrounding air. So Equation (34) can be simplified to:

(35) $$\left\{ \begin{array}{c} P=M\overset{˙}{p_{c}}=M{\left[{\overset{˙}{x}}_{c}\;\overset{˙}{y_{c}}\;\overset{˙}{z_{c}}\right]}^{T}\\ L=p_{c}\times M\overset{˙}{p_{c}} \end{array}\right.$$

Bring equation (35) into equation (33) to get the ZMP coordinate expression as:

(36) $$\left\{ \begin{array}{c} x_{p}=x_{c}+\frac{(z_{p}-z_{c}){\overset{¨}{x}}_{c}}{g\cos\beta^{\prime }+{\overset{¨}{z}}_{c}}\\ y_{p}=y_{c}+\frac{\left(z_{p}-z_{c})({\overset{¨}{y}}_{c}+g\sin\beta^{\prime }\right)}{g\cos\beta^{\prime }+{\overset{¨}{z}}_{c}} \end{array}\right.$$

As shown in Fig. 8, according to the geometric size and position symmetry relationship of the food delivery robot, under the global coordinate system, the position coordinates of the center of gravity $p_{c}=[0\;0\;h]$. Since the global coordinate system z-axis is perpendicular to the motion slope, and the ZMP coordinate falls on the support slope, the $z_{p}=0$. Substitute the coordinates into equation (36) to obtain:

(37) $$\left\{ \begin{array}{c} x_{p}=-\frac{h{\overset{¨}{x}}_{c}}{g\cos\beta^{\prime }+{\overset{¨}{z}}_{c}}\\ y_{p}=-\frac{h({\overset{¨}{y}}_{c}+g\sin\beta^{\prime })}{g\cos\beta^{\prime }+{\overset{¨}{z}}_{c}} \end{array}\right.$$

When the takeaway delivery robot performs a climbing motion at a lateral uniform speed on the slope, the acceleration of the robot's center of gravity is 0 at this time. According to equation (37), the $x_{p}$ of the zero moment point is 0, and the only $y_{p}$ coordinates the ZMP point has are:

(38) $$y_{p}=-\frac{h(g\sin\beta^{\prime })}{g\cos\beta^{\prime }}$$

According to formula (37) and (38), combined with the robot's support polygon, it is convenient to determine whether the robot will be unstable. To measure the size of the robot's ability to maintain stability, the stability margin $S_{m}$ is used as the standard. As shown in Fig. 8, for a six-wheeled mobile robot, define the stability margin as the shortest distance from the ZMP to support the polygon boundary, i.e.:

(39) $$S_{m}=\min(S_{m1},S_{m2},S_{m3},S_{m4},S_{m5},S_{m6})$$

According to formula (39), when the food delivery robot moves at a lateral and uniform speed on the slope, at this time:

(40) $$S_{m}=\min(n-\frac{h{\overset{¨}{x}}_{c}}{g\cos\beta^{\prime }+{\overset{¨}{z}}_{c}},\frac{B}{2}-\frac{h({\overset{¨}{y}}_{c}+g\sin\beta^{\prime })}{g\cos\beta^{\prime }+{\overset{¨}{z}}_{c}})$$

The stability of the six-wheeled food delivery robot was analyzed by the zero-torque point method, and formulas (25) and (37) described the relationship between the angle formed between the left and right wheels and the ground formation of the left and right wheels and the stability margin of the robot when the left and right side wheels were not horizontal, and the performance of the food delivery robot was determined when the lateral movement of the ramp or the unilateral obstacle crossing of the left and right sides was determined. Analytical formula (40) can be seen that the factors affecting the stability margin of the food delivery robot mainly include the robot body width, the height of the robot's center of gravity, the acceleration of the robot's movement and the deviation angle generated by the shock absorption structure of the robot suspension The above theoretical derivation provides a theoretical basis for the design of the food delivery robot mechanism, slope motion control and obstacle crossing performance.

### 2.3 Non-horizontal stability analysis of the front and rear wheels

**Figure 9:**
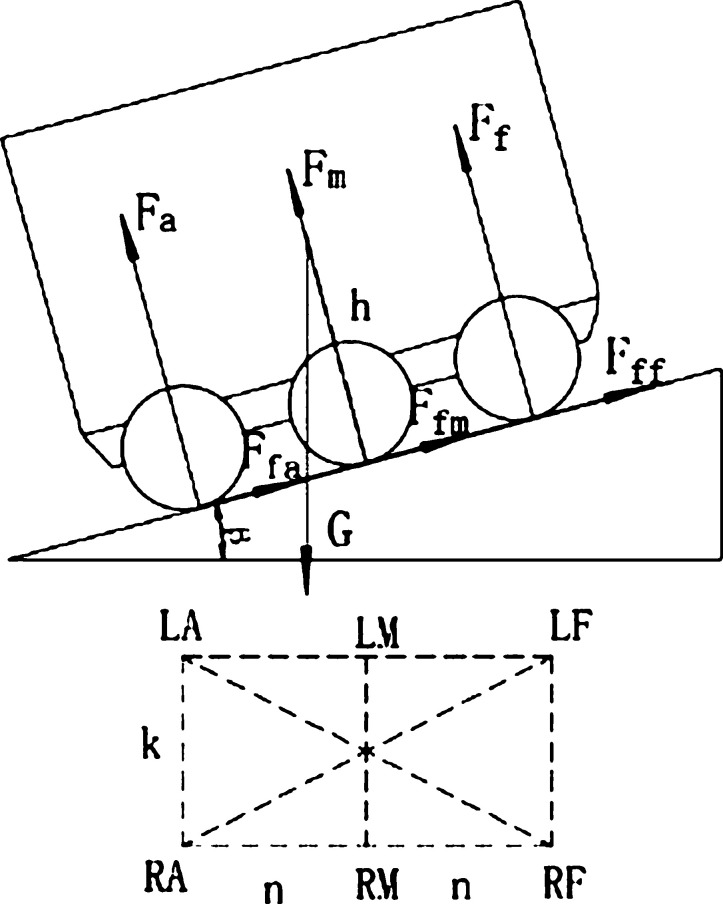
Stress analysis diagram of each wheel when the front and rear wheels move non horizontally.

Fig. 9 is the force analysis diagram of each wheel when the food delivery robot climbs a hill or passes the speed reduction belt, and the wheels on the front and rear sides of the food delivery robot are not horizontally moving. $F_{a}$ is the sum of the support forces of the ground facing the rear wheel; the $F_{fa}$ is the sum of the friction forces of the ground facing the rear wheel; the $F_{m}$ is the sum of the support forces of the ground facing the middle wheel; the $F_{fm}$ is the sum of the friction forces of the ground facing the middle wheel; the $F_{f}$ is the sum of the support forces of the ground facing the front wheel; and the $F_{ff}$ is the sum of the friction forces of the ground facing the front wheel.

The force analysis of the food delivery robot can obtain the supporting force of each wheel as follows:

(41) $$\left\{ \begin{array}{c} F_{f}=\frac{1}{6}G\cos\beta -\frac{Gh\sin\beta }{4L}\\ F_{m}=\frac{1}{6}\cdot G\cos\beta \\ F_{a}=\frac{1}{6}G\cos\beta +\frac{Gh\sin\beta }{4L} \end{array}\right.$$

When the robot runs smoothly on a flat road, ignoring the impact force caused by the bumps on the ground, the *F* in equation (7) is the support force of the robot. In the substitution type (41) (7), the displacement bias of the suspension shock absorption structure of the robot moving laterally on the slope surface is obtained:

(42) $$\left\{ \begin{array}{c} x_{f}=\frac{a^{2}}{2b^{2}k_{2}}\left(\frac{1}{6}\cdot G\cos\beta -m_{1}g-\frac{Gh\sin\beta }{4L}\right)\\ x_{m}=\frac{a^{2}}{2b^{2}k_{2}}\left(\frac{1}{6}\cdot G\cos\beta -m_{1}g\right)\\ x_{a}=\frac{a^{2}}{2b^{2}k_{2}}\left(\frac{1}{6}\cdot G\cos\beta -m_{1}g+\frac{Gh\sin\beta }{4L}\right) \end{array}\right.$$

When the food delivery robot travels laterally on the slope, the angle of the front and rear wheels relative to the horizontal plane $\beta^{\prime \prime }$ is the slope angle of the slope *β* and the tilt angle due to the suspension structure Δ*β* sum, that is:

$$\beta^{\prime \prime }=\beta +\Delta \beta$$

According to the formula (42), that is, the displacement bias of the front and rear wheels of the spring damping mechanism $x_{f}$ and $x_{a}$, it is possible to obtain:

(43) $$\sin\Delta \beta =\frac{Ga^{2}h\sin\beta }{4k_{2}b^{2}L^{2}}$$

When the value of the Δ*β* is small, Δβ=sin⁡Δβ, then, according to formula (43):

(44) $$\beta^{\prime \prime }=\beta +\frac{Ga^{2}h\sin\beta }{4k_{2}b^{2}L^{2}}$$

Taking the projection point of the center of mass perpendicular to the slope of the food delivery robot as the origin of the coordinate system, the global coordinate system is established, and the x-axis is specified to point the forward direction of the robot, the z-axis is perpendicular to the oblique face, and the y-axis is determined by the right-hand rule. In the global coordinate system, the gravity acceleration is set to $g^{\prime }=[-g\sin\beta^{\prime \prime }\;0-g\cos\beta^{\prime \prime }]$, $\beta^{\prime \prime }$ is the angle between the front and rear wheels and the horizontal plane of the food delivery robot under the influence of the spring shock absorption structure, and the center of gravity coordinate is $p_{c}=[x_{c}\;y_{c}\;z_{c}]$; $p^{\prime }$ point is the position where ZMP is located, the coordinates are $p^{\prime }=[x_{p}\;y_{p}\;z_{p}]$, and the support point is $f^{\prime }$ by the ground reaction force; the moment $\tau^{\prime }$ of the ground reaction force around the origin is:

(45) $$\tau^{\prime }=p^{\prime }\cdot f^{\prime }+\tau_{p}^{\prime }$$

where: $\tau_{p}^{\prime }$ is the moment at the zero-crossing moment point.

Derived from the momentum theorem and the angular momentum theorem:

(46) $$\overset{˙}{P}=Mg^{\prime }+f^{\prime }$$

(47) $$\overset{˙}{L}=p_{c}\times Mg^{\prime }+\tau^{\prime }$$

Syndicates (45), (46) and (47), eliminating *τ* and *f* yields:

(48) $$\tau_{p}^{\prime }=\overset{˙}{L}-p_{c}\times Mg^{\prime }+(\overset{˙}{P}-Mg^{\prime })\times p^{\prime }$$

From ZMP theory, the horizontal components $\tau_{px}^{\prime }$ and $\tau_{py}^{\prime }$ in the equation (48) are 0, and the ZMP position coordinates are solved $x_{p}$ and $y_{p}$ are:

(49) $$\left\{ \begin{array}{c} x_{p}=\frac{x_{c}\cdot Mg^{\prime }\cos\beta^{\prime \prime }+z_{p}({\overset{˙}{P}}_{x}+{\mathrm{Mg}}^{\prime }\sin\beta^{\prime \prime })-z_{c}\cdot Mg^{\prime }\sin\beta^{\prime \prime }-{\overset{˙}{L}}_{y}}{Mg\cos\beta^{\prime }+{\overset{˙}{P}}_{z}}\\ y_{p}=\frac{{\overset{˙}{L}}_{x}+y_{c}\cdot Mg^{\prime }\cos\beta^{\prime \prime }+{\overset{˙}{P}}_{y}z_{p}}{{\overset{˙}{P}}_{z}+{\mathrm{Mg}}^{\prime }\cos\beta^{\prime \prime }} \end{array}\right.$$

The takeaway delivery robot is a quality point system, so its momentum and angular momentum are:

(50) $$\left\{ \begin{array}{c} P=\sum_{i}P_{i}=\sum_{i}m_{i}{\overset{˙}{p}}_{i}\\ L=\sum_{i}L_{i}=\sum_{i}p_{i}\times m_{i}{\overset{˙}{p}}_{i} \end{array}\right.$$

The takeaway delivery robot runs at a uniform speed when delivering food, and when moving on the slope, in order to ensure the safety of the takeaway robot, only the straight motion is carried out, ignoring the influence of the surrounding air. So equation (50) can be simplified to:

(51) $$\left\{ \begin{array}{c} P=M\overset{˙}{p_{c}}=M{\left[{\overset{˙}{x}}_{c}\;\overset{˙}{y_{c}}\;\overset{˙}{z_{c}}\right]}^{T}\\ L=p_{c}\times M\overset{˙}{p_{c}} \end{array}\right.$$

Bring equation (51) into equation (49) and get the ZMP coordinate expression as:

(52) $$\left\{ \begin{array}{c} x_{p}=x_{c}+\frac{(z_{p}-z_{c}){\overset{¨}{x}}_{c}+(z_{p}-z_{c})Mg\sin\beta^{\prime \prime }}{g\cos\beta^{\prime \prime }+{\overset{¨}{z}}_{c}}\\ y_{p}=y_{c}+\frac{(z_{p}-z_{c}){\overset{¨}{y}}_{c}}{g\cos\beta^{\prime \prime }+{\overset{¨}{z}}_{c}} \end{array}\right.$$

**Figure 10:**
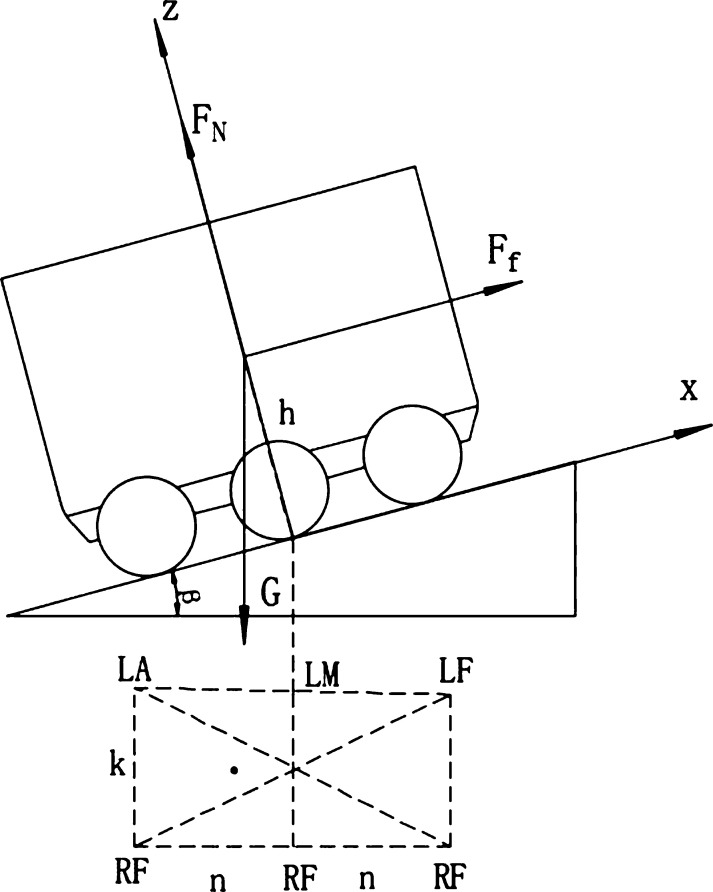
ZMP determination method when the front and rear wheels are not horizontal.

As shown in Fig. 10, the geometric dimensions and position symmetry of the food delivery robot are known, and in the global coordinate system, the position coordinates of the center of gravity $p_{c}=[0\;0\;h]$. Since the global coordinate system z-axis is perpendicular to the motion slope, and the ZMP coordinate falls on the support slope, the $z_{p}=0$. Substitute the coordinates into equation (52) to obtain:

(53) $$\left\{ \begin{array}{c} x_{p}=-\frac{h{\overset{¨}{x}}_{c}+hg\sin\beta^{\prime \prime }}{g\cos\beta^{\prime \prime }+{\overset{¨}{z}}_{c}}\\ y_{p}=\frac{-h{\overset{¨}{y}}_{c}}{g\cos\beta^{\prime \prime }+{\overset{¨}{z}}_{c}} \end{array}\right.$$

When the takeaway delivery robot performs a climbing motion at a uniform speed on the slope, the acceleration of the robot's center of gravity is 0 at this time. According to equation (53), the $y_{p}$ of the zero moment point is 0, and the ZMP point has only $x_{p}$ coordinates are:

(54) $$x_{p}=-\frac{hg\sin\beta^{\prime \prime }}{g\cos\beta^{\prime \prime }}$$

According to formula (53) and (54), combined with the robot's support polygon, it is easy to determine whether the robot will be unstable. As shown in Fig. 7, for a food delivery robot, the stability margin is the shortest distance from the ZMP to support the polygon boundary, i.e.:

(55) $$S_{m}=\min(S_{m1},S_{m2},S_{m3},S_{m4},S_{m5},S_{m6})$$

The takeaway food delivery robot climbs at a uniform speed on the slope, according to formula (55) at this time:

(56) $$S_{m}=\min(\frac{B}{2}-\frac{h{\overset{¨}{y}}_{c}}{g\cos\beta^{\prime \prime }+{\overset{¨}{z}}_{c}},L-\frac{h{\overset{¨}{x}}_{c}+hg\sin\beta^{\prime \prime }}{g\cos\beta^{\prime \prime }+{\overset{¨}{z}}_{c}})$$

During the movement of the food delivery robot, when the ZMP is inside the support polygon, the $S_{m}>0$, the robot is in a stable state; when the ZMP falls outside the support polygon, the $S_{m}<0$, and the robot may tip over. The larger the stability margin $S_{m}$ value, the more stable the robot becomes. When ZMP is located in the center of the support polygon, that is, when the robot is moving on the horizontal plane, the stability margin of the robot is the greatest and the robot is the most stable.

The stability of the six-wheeled food delivery robot was analyzed by mechanical analysis and zero torque point method. Formula (44) and formula (56) expressed the relationship between the angle formed between the front and rear wheels and the ground and the stability margin of the robot when the front and rear side wheels were not horizontal. The performance of the food delivery robot was determined when the longitudinal movement of the ramp or the front and rear side wheels crossed the obstacle. It can be seen from the analysis (56) that the factors affecting the stability margin of the food delivery robot mainly include the length of the robot's body, the height of the robot's center of gravity, the acceleration of the robot's movement, and the deviation angle generated by the front and rear side wheels of the robot suspension shock absorption structure, which provides a theoretical basis for the design of the food delivery robot mechanism, slope motion control and obstacle crossing performance.

## 3 Simulation analysis of food delivery robots

**Figure 11:**
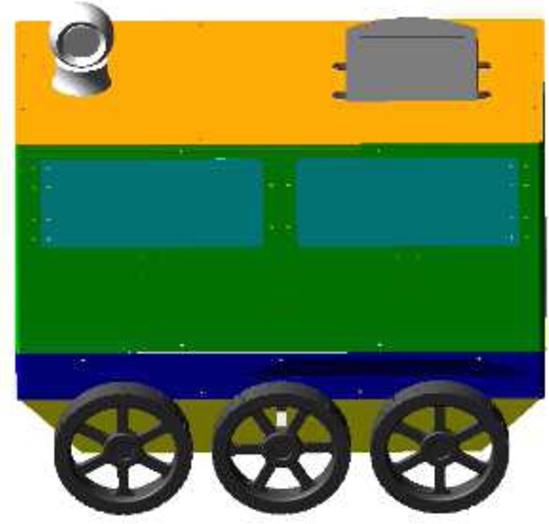
Model diagram of meal delivery robot under Adams.

Fig. 11 shows the model diagram of the food delivery robot under ADAMS, the length of the robot is 800 mm, the width is 400 mm, and the height is 610 mm; the spacing between the front, middle and rear wheels of the robot is 250 mm; the wheel distance between the left and right sides is 420 mm, and the diameter of the wheel is 200 mm; the length of the spring shock absorber of the suspension shock structure is 100 mm, the length of the motor and shock absorber is 135 mm, and the angle of the spring shock absorber and the horizontal plane is 55°.

### 3.1 Kinematics and dynamics simulation analysis

Using ADAMS simulation software, the kinematics and dynamics of the food delivery robot during steering are simulated, and the movement process is divided into linear motion - turn 90° - linear motion.

In the motion simulation process of the food delivery robot, the food delivery robot first performs a linear movement, and the speed of the left and right wheels is increased from 0 to 110 rpm. Then, the speed of the right wheel is increased while the left wheel speed of the fixed robot is unchanged, and the food delivery robot turns to the left. Finally, reduce the speed on the right side of the food delivery robot so that the left and right wheel speeds are equal to 110 rpm, and the linear movement is carried out again.

**Figure 12:**
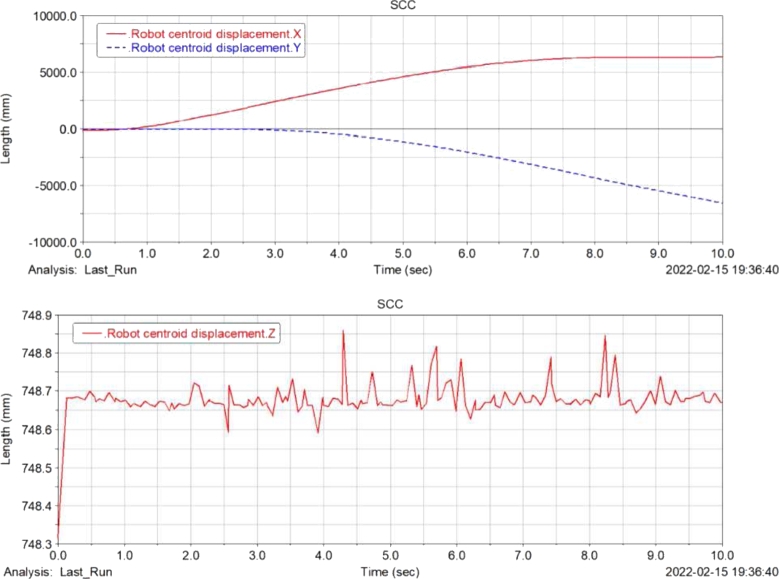
Displacement curve of centroid of meal delivery robot in X, y and Z directions.

**Figure 13:**
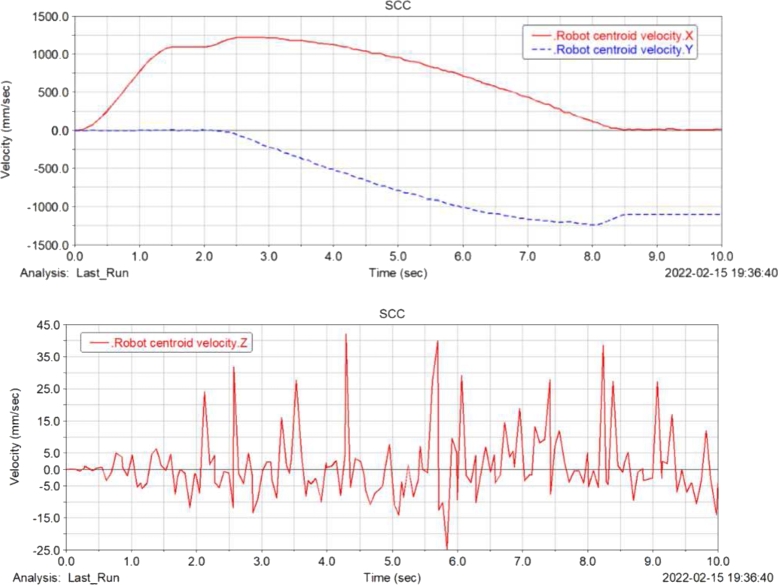
Speed curve of centroid of meal delivery robot in X, y and Z directions.

**Figure 14:**
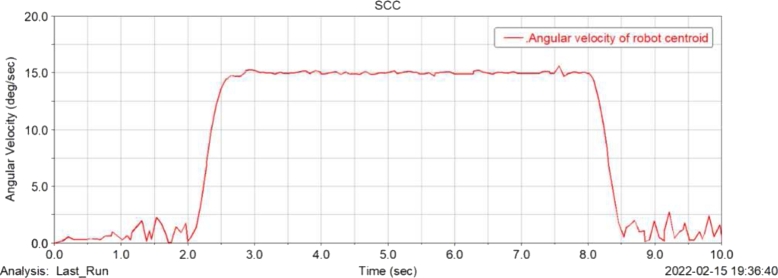
Angular velocity curve of centroid of meal delivery robot.

**Figure 15:**
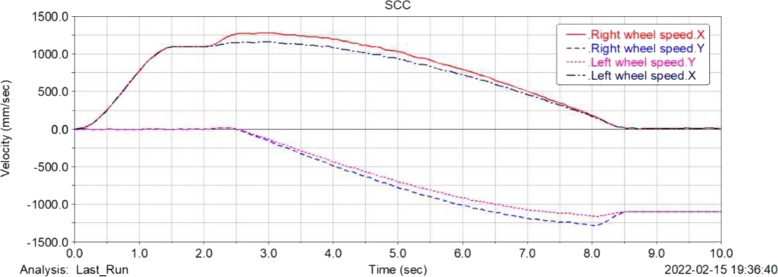
Speed comparison diagram of left and right wheels of meal delivery robot in X and Y directions.

Figure 12, Figure 13, Figure 14, Figure 15(From ADAMS) show the displacement, velocity curve and angular velocity curve of the food delivery robot centroid in X, Y, Z directions. In the figures, the maximum fluctuation of the robot in the Z axis direction is 0.25 mm, and the maximum fluctuation of the speed range is 65 mm/s, indicating that the robot is still relatively stable after the suspension shock absorption structure, and there will not be too much fluctuation; the displacement and speed in the X and Y axis directions are linear changes, the change is relatively gentle, and almost no offset occurs, indicating that the robot is relatively stable when moving, and there will be no large slip phenomenon.

The analysis of the four curves can be seen that after the robot moves in a straight line, changing the speed of one of the wheels can make the robot move more smoothly. At this time, the robot's displacement, speed and angular velocity curves are relatively smooth, and no large fluctuations occur, which verifies the kinematic characteristics of the food delivery robot and provides a certain reference value for the subsequent structural design and optimization of the food delivery robot.

**Figure 16:**
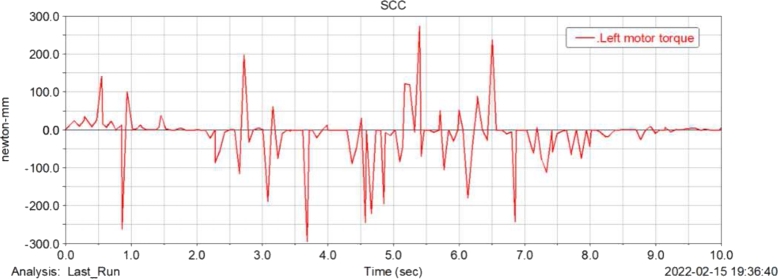
Torque simulation diagram of left drive wheel motor of meal delivery robot.

**Figure 17:**
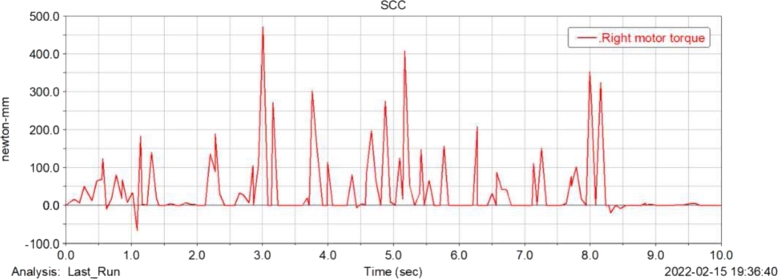
Torque simulation diagram of right drive wheel motor of meal delivery robot.

Figure 16, Figure 17(From ADAMS) show the torque simulation diagram of the left and right drive wheel motor of the food delivery robot, through the analysis, the maximum torque output of the motor at this time is 470 N/mm, according to the structural design of the previous article, the motor reducer can provide 637 N/mm of torque, so the power design of the robot when turning meets the actual design requirements.

**Figure 18:**
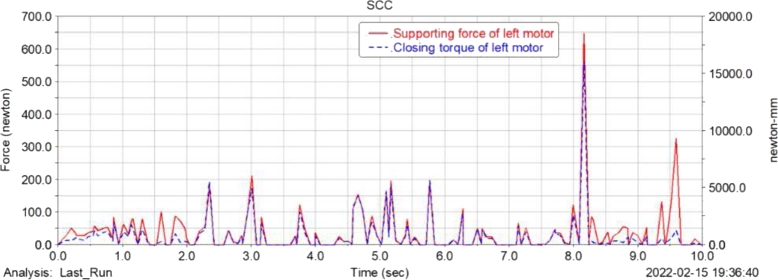
Change curve of supporting force and closing torque of robot left wheel motor.

**Figure 19:**
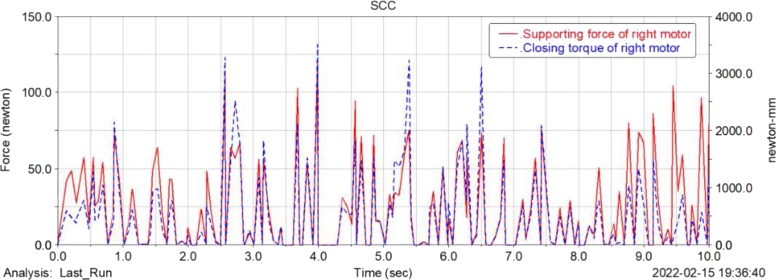
Change curve of supporting force and closing torque of robot right wheel motor.

Figure 18, Figure 19(From ADAMS) show the support force change curve and the change curve of the received force moment of the left and right wheel motors of the food delivery robot. As shown in the figure, the left and right wheel motors and motor supports of the food delivery robot are subject to greater support forces and torques during the turning process, and the support force and the received moments fluctuate greatly during the movement. Therefore, the design needs to further consider the bearing capacity of the motor bracket during the movement and strengthen the strength of the structural parts in the corresponding position.

### 3.2 Stability simulation analysis

In order to verify the stability of the takeaway delivery robot and the shock absorption effect of the suspension shock absorption structure, the food delivery robot was simulated in the ADAMS when it moved on the slope, slope lateral movement, and over the speed bump. By comparing and simulating the mobile robot with both large stiffness suspension structure and the small stiffness suspension shock absorption structure, the shock absorption effect of the suspension shock absorption structure and its influence on the stability of the robot when climbing, ramping laterally and crossing the speed reduction belt are obtained. According to the size parameters of the model prototype, the parameters such as the material of the model and the stiffness of the spring are set in the ADAMS software, and the simulation experiments are carried out on the robot in different environments.

(1) Stability simulation when passing the speed bump

**Figure 20:**
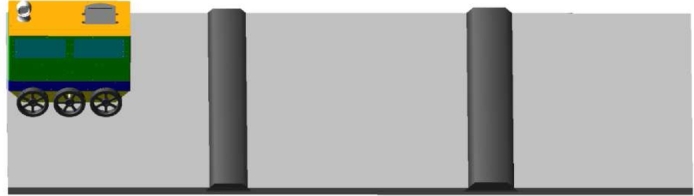
Simulation diagram of robot passing through deceleration belt.

Fig. 20 shows the ARAMS simulation analysis of the takeaway delivery robot when passing the speed reduction belt, the height of the two speed reduction belts is 60 mm and 70 mm, and the slope angle of the slope is 45 degrees. The stiffness of the spring is changed to verify the displacement fluctuation and speed fluctuation at the center of mass when the robot passes the speed reduction belt under different spring stiffnesses.

When the takeaway food delivery robot passes the speed bump, the stiffness of the spring shock absorber is set at 20 N/m, 30 N/m, 40 N/m, 60 N/m, 90 N/m, and 500 N/m, respectively. Comparative analysis of curves under different stiffnesses shows that, when the spring stiffness is relatively low, the displacement and speed fluctuations at the robot center of mass are relatively gentle through the speed reduction belt, with a good shock absorption effect. When the spring stiffness is too low, the fluctuation of the displacement and speed at the robot centroid is relatively flat, but the range of fluctuations is larger; when the spring stiffness is too large, its displacement curve changes very steeply when entering and leaving the speed reduction belt with a larger slope, and the shock absorption effect is poor. Comparing several sets of data, it is found that when the spring stiffness is 40 N/m, the deceleration effect of the robot is better, and the spring shock absorber will not reach the incremental limit, and the displacement fluctuation range is also more appropriate.

**Figure 21:**
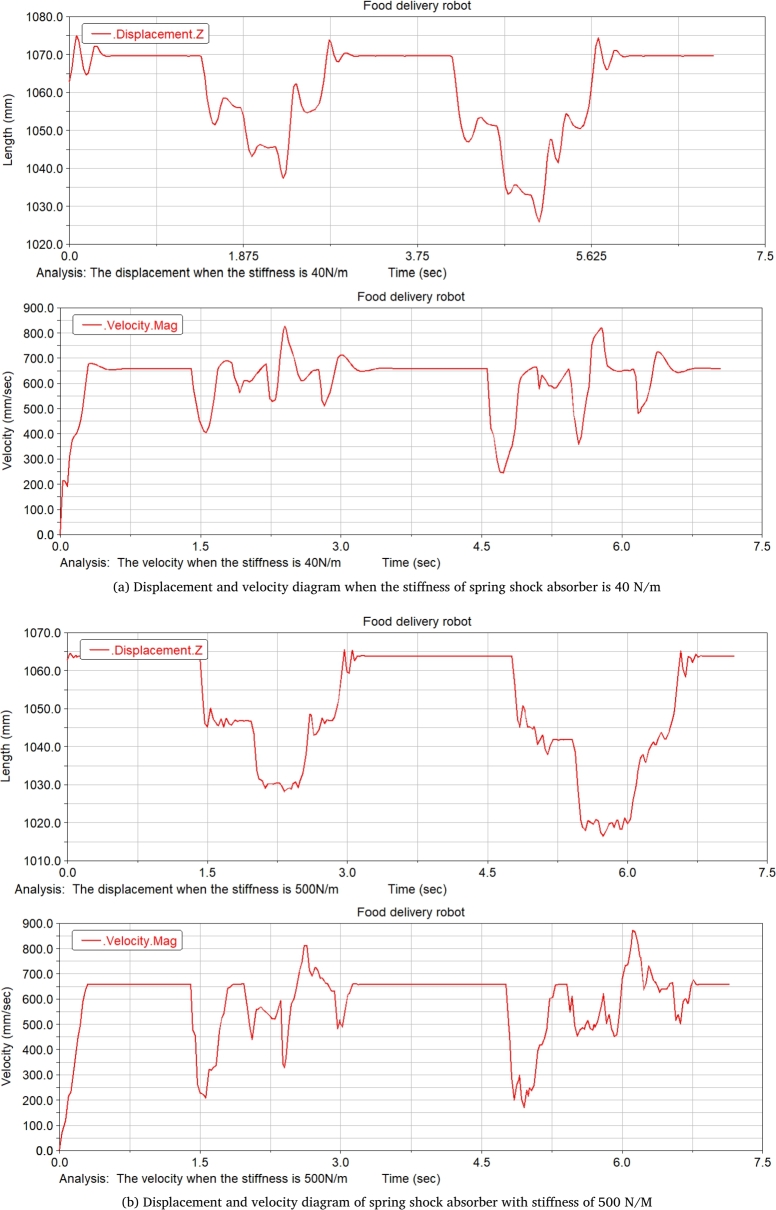
Displacement and velocity curves at the center of mass under different spring shock absorber stiffness.

Fig. 21(From ADAMS) shows the displacement and velocity curves at the center of mass of the food delivery robot at a stiffness of 40 N/m and 500 N/m.

During the simulation analysis, it was found that when the stiffness of the spring shock absorber is increased continuously, and the height of the speed reduction belt is increased appropriately, various unexpected phenomena, such as slippage, overturning or insufficient power caused by the wheel off the ground. Analyzing the causes of the phenomena, when the slope of the speed reduction belt is large, and the spring is just too large, the front wheel or rear wheel may be off the ground when the robot passes through the speed reduction belt because of the absence of good shock absorption, so that the robot center of gravity is unstable or insufficient power, resulting in various accidents. Therefore, the suspension shock absorption structure with the right stiffness has a good auxiliary effect when the robot passes the speed reduction belt, and has a good alleviating effect on various unexpected situations.

(2) Stability simulation when climbing

**Figure 22:**
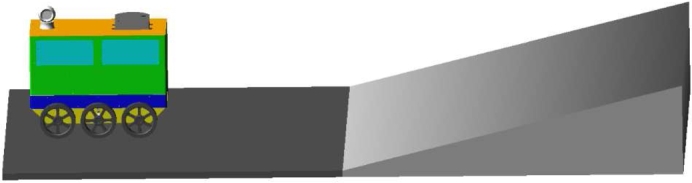
Simulation diagram of robot climbing.

Fig. 22 shows a simulation of a takeaway delivery robot climbing a hill. By changing the gradient and the stiffness of the spring shock absorber, the change and difference in the length of the front and rear wheel spring shock absorbers at different slopes were obtained. The simulation experiment of the support force change of the front wheel and the slope of the slope when the tilt occurred was carried out under the condition of two spring stiffnesses of 40 N/m and 400 N/m.

**Figure 23:**
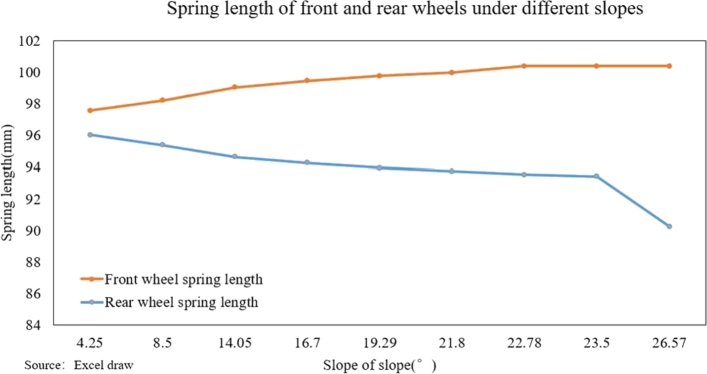
Variation curve of front and rear wheel spring length under different slopes.

Fig. 23(From ADAMS) shows the curve of the change in the length of the front and rear wheel springs at different slopes. It can be seen from the figure that with the increase of the slope of the slope, the spring length of the front wheel gradually increases until it reaches the limit length of the spring; the spring length of the rear wheel gradually shortens with decreasingly rate of shortening. As the slope of the slope increases, the spring length difference between the front and rear wheels gradually increases, which will cause the robot to produce a certain angle with the support surface and reduce the stability of the robot.

**Figure 24:**
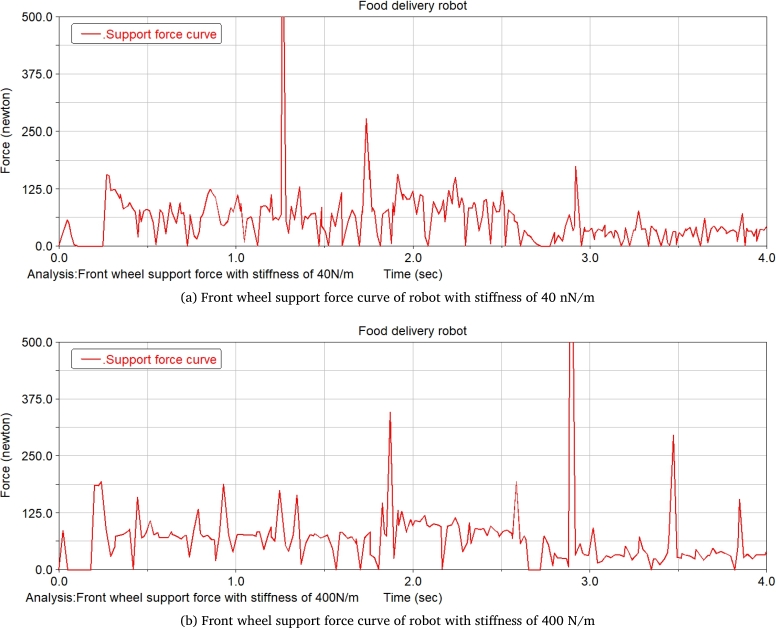
Front wheel support force curve of meal delivery robot when the slope is 14.05°(From ADAMS).

**Figure 25:**
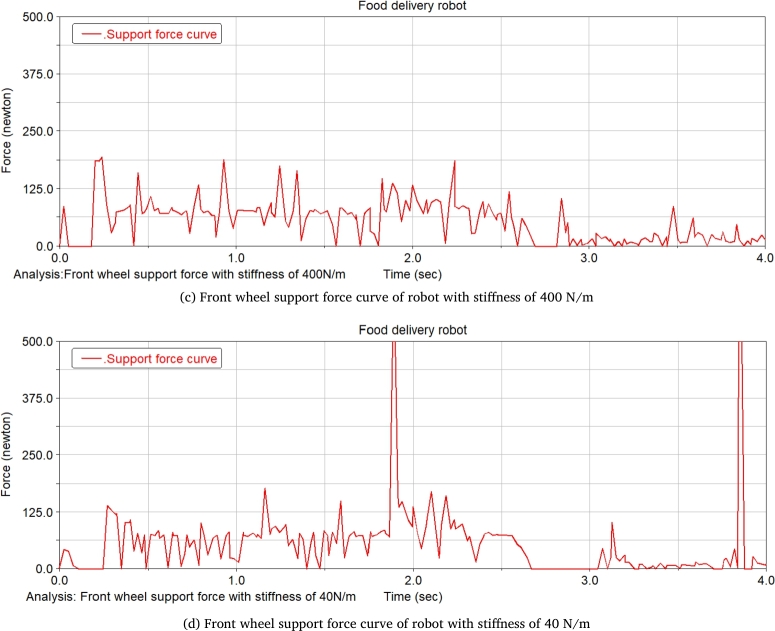
Front wheel support force curve of meal delivery robot when the slope is 21.8°(From ADAMS).

The food delivery robot is simulated at two spring rates of 40 N/m and 400 N/m, which changes the slope of the slope. The change of the front wheel support force of the food delivery robot is recorded. Fig. 24 shows the front wheel support force curve of the food delivery robot with a slope of 14.05°. It can be seen from the figure that when the robot moves on the slope, the support force of the front wheel of the robot with a stiffness of 40 N/m is significantly reduced. Increasing the degree of the slope till 21.8°, the robot with a stiffness of 400 N/m does not have an obvious support force for the 0 stage, and the robot with a stiffness of 40 N/m obviously has a stage of support force of 0 at one end, and with the increase of the slope, the support force of 0 will increase until it is unstable, and its front wheel support force is shown in Fig. 25.

**Figure 26:**
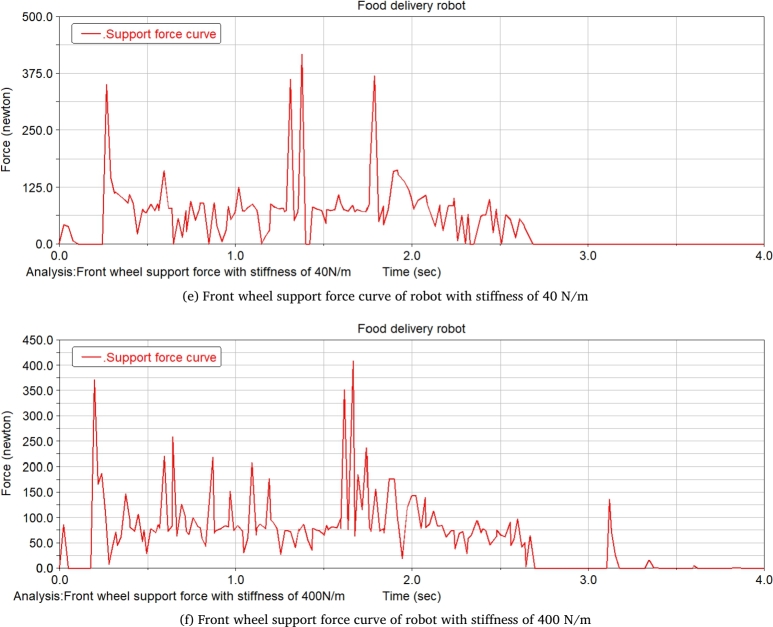
Front wheel support force curve of meal delivery robot when the slope is 26.79°(From ADAMS).

**Figure 27:**
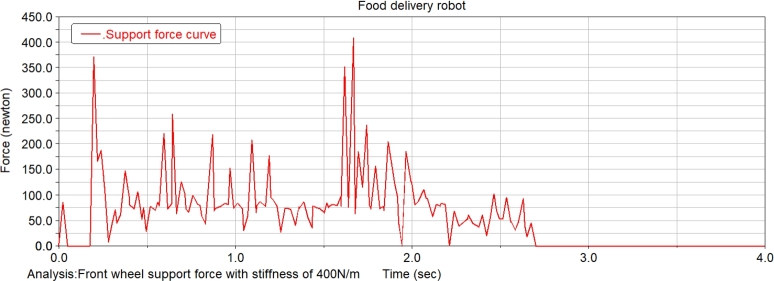
Front wheel support force curve of robot with spring stiffness of 400 N/m when the slope is 29.03°(From ADAMS).

After the stage of support force 0, the slope of the slope is constantly changed for simulation, when the slope of the slope reaches 26.79°, the robot with a stiffness of 40 N/m will be unstable and overturned, while the robot with a stiffness of 400 N/m can continue to run, and the support force is shown in Fig. 26. Continues changing the degree of the slope till 29.03°, the robot with a stiffness of 400 N/m will have an unstable tilt, and its support force is shown in Fig. 27.

### 3.3 Experimental verification

**Figure 28:**
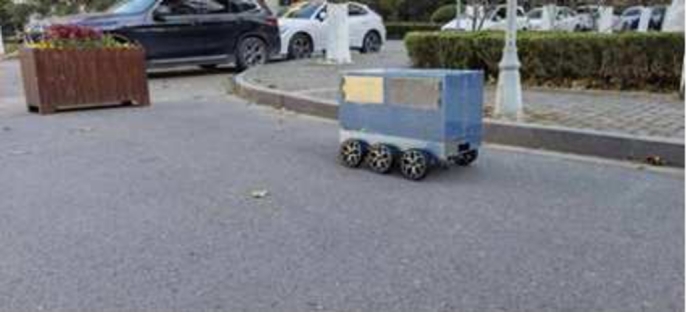
Normal driving A.

**Figure 29:**
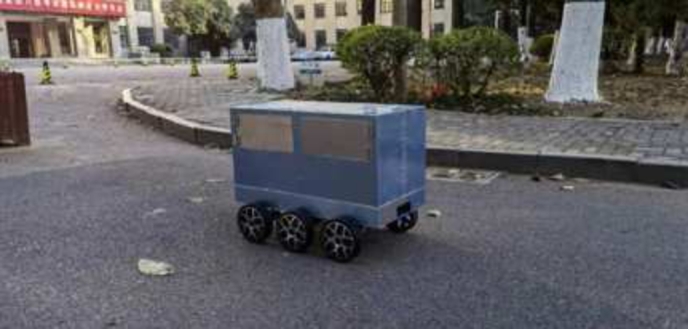
Normal driving B.

**Figure 30:**
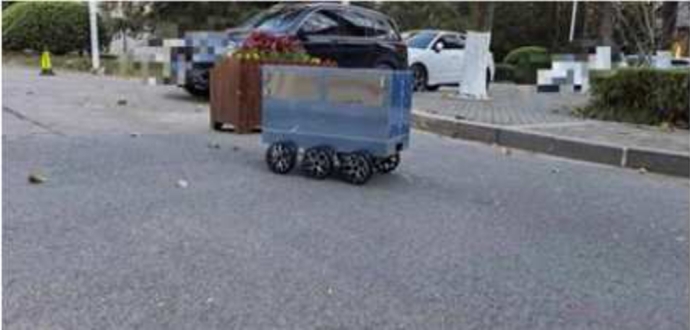
Approaching obstacles.

**Figure 31:**
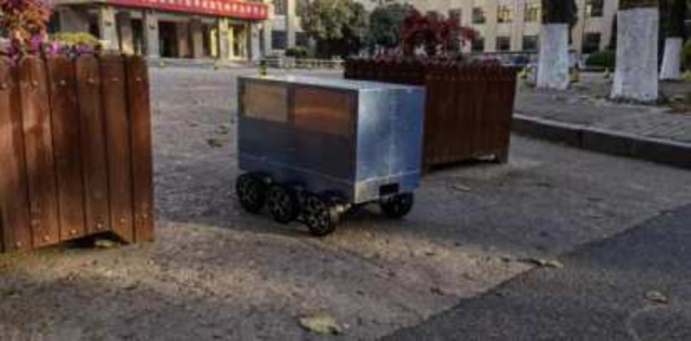
Avoiding and crossing obstacles.

Between April and May 2022, we conducted some functional tests of the corresponding food delivery robots in the campus environment, including road experiments such as normal driving (Figure 28, Figure 29, Figure 30, Figure 31), crossing obstacles and avoiding obstacles under the normal conditions of the robots on campus roads. The experiments verified the corresponding functions of the six-wheel outdoor food delivery robot we designed, laying the foundation for further functional verification in the future.

## 4 Results and discussion

The research on takeaway food delivery robots is self-evident in the current pandemic. The paper presents the independent suspension structure of the food delivery robot we studied. Based on the independent suspension structure, the Lagrangian equation is used to analyze the dynamics of the suspension damping structure of the food delivery robot to obtain the wheel offset of the wheel under different driving conditions. After deriving the additional tilt angle caused by the suspension damping structure, the zero-moment point method is used to analyze the stability in the two sports environments. This method is also applicable to the stability analysis of other mobile robots with suspension damping structures. The ADAMS simulation analysis process is:

(1) When passing the speed bump, comparing the curve analysis under spring stiffness of 20 N/m, 30 N/m, 40 N/m, 60 N/m, 90 N/m, 500 N/m, it is found that when the spring stiffness is relatively low, the robot can pass through the deceleration belt with a good shock absorption effect; When the spring stiffness is too low, the fluctuation of the displacement and velocity at the center of mass of the robot is gentle, and the fluctuation range is larger; When the spring stiffness is too large, the curve changes very steeply with a larger slope when the robot enters and leaves the speed bump, and the shock absorption effect is poor; Comparing several sets of data, it is found that when the spring stiffness is 40 N/m, the deceleration effect of the robot is better, and the spring shock absorber will not reach the incremental limit, and the displacement fluctuation range is more appropriate; Suspension damping structure with appropriate stiffness has a good auxiliary effect for the robot to pass through the speed bump, and has a good alleviation effect on various accidents.
(2) Simulation analysis of food delivery robot climbing slope. Under the two spring stiffnesses of 40 N/m and 400 N/m, the food delivery robot was simulated, the slope of the inclined plane was changed, and the changes in the support force of the front wheels of the food delivery robot were observed. When moving on an inclined surface with a slope of 14.05 degrees, the support force of the front wheel of the food delivery robot with a stiffness of 40 N/m is significantly reduced; When the slope of the inclined plane is 21.8 degrees, the food delivery robot with a stiffness of 400 N/m has no obvious support force at stage 0, and the food delivery robot with stiffness of 40 N/m obviously has a stage where one end support force is 0. As the slope increases, the time when the supporting force is 0 will increase until it loses stability; After the stage where the supporting force is zero, the slope of the inclined plane is continuously changed for simulation. When the slope of the inclined plane reaches 26.79 degrees, the food delivery robot with a stiffness of 40 N/m will overturn, and the food delivery robot with a stiffness of 400 N/m can continue to run. Continue to change the slope of the slope. When the slope of the slope is 29.03 degrees, the food delivery robot with a rigidity of 400 N/m will fall over.
(3) The simulation results prove that the food delivery robot with an independent suspension structure has a better shock absorption effect under appropriate spring stiffness; experiment verifies the rationality of the structure design of the food delivery robot with a suspension damping structure and the correctness of the theoretical analysis.

There is still a certain gap between the simulation experiment environment of the thesis and the real complex road environment. Therefore, we plan to carry out the experimental verification of the food delivery robot in the field environment. The research work done in this paper laid a certain foundation for minimizing the impact of suspension damping on the stability of the robot. The stability analysis of a food delivery robot with a suspension damping structure provides a theoretical basis for the study of mobile robots with a flexible structure and the stability analysis in a complex terrain environment.

## CRediT authorship contribution statement

### Author contribution statement

**Jiang Shuhai:** Conceived and designed the experiments; Performed the experiments; Analyzed and interpreted the data; Contributed reagents, materials, analysis tools or data; Wrote the paper.

**Song Wei:** Performed the experiments; Analyzed and interpreted the data.

**Zhou Zhongkai, Sun Shangjie:** Contributed reagents, materials, analysis tools or data; Wrote the paper.

### Funding statement

Director Shuhai Jiang was supported by The National Special Research Fund for Non-profit Sector [201404402-03].

### Data availability statement

Data included in article/supp. material/referenced in article.

### Declaration of interests statement

The authors declare no conflict of interest.

### Additional information

No additional information is available for this paper.
